# Influence of Extracellular Mimicked Hierarchical Nano-Micro-Topography on the Bacteria/Abiotic Interface

**DOI:** 10.3390/polym12040828

**Published:** 2020-04-05

**Authors:** Sílvia Ferreira, Ana P. Piedade

**Affiliations:** University of Coimbra, CEMMPRE, Department of Mechanical Engineering, 3030-788 Coimbra, Portugal; silviamferreira94@gmail.com

**Keywords:** nano-micro-wrinkles, hierarchical topographies, mimic extracellular matrices, polyamide thin films, prokaryotic cells

## Abstract

The study of interfaces between engineered surfaces and prokaryotic cells is a subject whose actual relevance has been reinforced by the current outbreaks due to unknown viruses and antibiotic-resistant bacteria. Studies aiming at the development of antibacterial surfaces are based on two pillars: surface chemistry or topographical cues. This work reports the study of only the topographic aspect by the development of thin films of polyamide, which present attractive surface chemistry for bacterial adhesion. The same chemistry with only nano- or hierarchical nano- and micro-topography that mimics the extracellular matrix is obtained by sputter-depositing the thin films onto Si and polydimethylsiloxane (PDMS), respectively. The surface average roughness of the Si-modified surfaces was around 1 nm, while the hierarchical topography presented values from 750 to 1000 nm, with wavelengths and amplitudes ranging from 15–30 µm and 1–3 µm, respectively, depending on the deposition parameters. The surface topography, wettability, surface charge, and mechanical properties were determined and related to interface performance with two Gram+ and two Gram- bacterial strains. The overall results show that surfaces with only nano-topographic features present less density of bacteria, regardless of their cell wall composition or cell shape, if the appropriate surface chemistry is present.

## 1. Introduction

Modeling surface chemistry and topography is necessary to ensure the success of the interaction between abiotic and biotic materials [1]. The complementary effect of each one of the characteristics/properties, provided they are well selected, will ensure successful application. Nevertheless, the final application and the type of cells that are going to interact with the synthetic material must always be considered. For eukaryotic cells, the success can, in fact, be achieved through adhesion, spreading, and proliferation [2,3], while for prokaryotic cells, an optimized surface can imply the ability to prevent adhesion, such as the case of antimicrobial surfaces [4].

Polymeric materials are easier to modify due to their properties and available processing techniques, which become advantageous for their use in several areas, including for biomedical applications [5]. Nevertheless, some polymers need long routes to be chemically modified in order to present the appropriate surface chemistry for interaction with biological compounds, an approach that has been used for a long time [6,7,8]. 

Considering the topographic features of a surface, currently, there are various techniques for changing the surface at the micro- and nano-scale. Some of these techniques are molding, nano/microprinting, laser ablation, and photolithography [9]. Among the different types of patterns that can be induced on a surface, wrinkles have emerged as a way of mimicking extracellular matrices due to the possibility of the spatial distribution of smooth, regular, and anisotropic architectures [10]. The wrinkles approach mimics the surfaces found in nature [11,12] in opposition to the engineered surfaces with grooves, pits, pillars, or channels often reported in literature [13].

Within the polymer materials, elastomers are preferred as most advantageous for the formation of surface wrinkles due to their low modulus of elasticity and, consequently, greater susceptibility to deformations induced by external stimuli [14,15]. The formation of wrinkled surfaces through the use of sputtering is not often reported in literature. Nonetheless, this physical-based technology, mainly the radio frequency (r.f.) magnetron mode, allows the deposition of any material, insulating or not, onto any surface. It is very versatile, for example, allowing the deposition of functionally graded hybrid thin films of metals and polymer [16], nanocomposite hybrid coatings [17], or preferentially oriented crystallographic metals [18]. Additionally, it is considered a green technique with zero-waste production, and, therefore, a sustainable engineering technology [19]. 

For wrinkle formation, the reported work in literature only describes the deposition of other classes of materials, such as metals [20,21] onto the surface of polymers. The formation of wrinkles can derive from the different linear thermal expansion coefficient between substrate and coating. An example of this is the use of r.f. magnetron sputtering for depositing silicon oxide on a substrate of poly(methylmethacrylate) [22]. The research demonstrated that the wrinkle formation occurred during the first stages of the deposition procedure. In fact, a thickness up to 10 nm was the minimal thickness necessary for obtaining a continuous film according to the authors, and the roughness increased but maintained its value for higher deposition times. In another work [23], the same technique was used for the deposition of tin-doped indium oxide (ITO) film onto a polydimethylsiloxane (PDMS) substrate. The obtained film had 40 nm thickness and a wrinkled surface. From the real-time recording of the process, the researchers found that the formation of the rough surface occurred between 5 to 10 s after the deposition started. This was the time that it corresponded to a thickness of the deposited film of 0.4 to 0.8 nm, which is not enough for the coalescence of the deposited material to form a continuous coating. Therefore, it was concluded that the deposited thin film was not responsible for the formation of the wrinkled surface. 

Nonetheless, the report of wrinkle formations by sputtering, due to the deposition of polymeric material onto a polymeric substrate, could not be found, which is the approach described in this work. In this manuscript, we report the deposition of poly(amide) 6.6 (PA) onto PDMS and silicon (Si) substrates in order to obtain the same surface chemistry, but with a different scale of surface topography. The wrinkle formation mechanisms are studied and, after abiotic characterization, the surfaces are tested in vitro with Gram-positive and Gram-negative bacteria to evaluate the microorganism adhesion. This procedure will allow us to determine the influence of topographic cues independently from the chemical ones in the response of the studied bacterial strains. 

The novelty of the reported work is to evaluate the real contribution of a nature mimicked hierarchical surface topography on the interface with bacteria, in opposition to groves and pits, which are engineered surfaces that also study the interaction with prokaryotic cells. Moreover, the process of surface modification by the deposition of a polymer onto a polymeric substrate eliminates the interface problems that induce coating failure and detachment.

The present study unequivocally aims to evaluate the influence of the topographic cues only in the antimicrobial activity must-use surfaces with an appropriate chemical composition to promote bacterial adhesion. Only in these terms can the topography be evaluated as an independent factor. 

## 2. Experimental Section

### 2.1. Surface Modification

The poly(amide) 6.6 thin films were deposited by radio frequence (r.f.) magnetron Edwards E306A sputtering equipment from a PA target (Goodfellow, Huntingdon, UK, 99,99% purity, diameter 100 mm), using Argon (Ar, 99.9999%) as the discharge gas. The substrates, PDMS (Sylgard^®^ 184, Dow Corning, Madrid, Spain, prepared according to manufacturer instructions) with a thickness of 400 mm and Si (polished, orientation (100) from Prolog Semicor Ltd., Kiev, Ukraine), were cut to 20 × 20 mm^2^ and were ultrasonically cleaned with alcohol and deionized water, for 10 min in each liquid, dried under hot air flow, before being introduced in the sputtering equipment. The ultimate pressure before each deposition was of 2.0 × 10^−4^ Pa, and the substrates and target were cleaned, prior to the deposition, with a shutter positioned between both in order to avoid cross-contamination. The cleaning process was made during 600s, with a power of 150, 200, and 250 W for the substrates (surfaces N1, N2, and N3, respectively) and 50 W for the target. The PA thin films were deposited, in non-reactive mode, with 60 W power during 900 s, at a pressure of 0.7 Pa. 

### 2.2. Surface Topography and Morphology

The coated PDMS surfaces were characterized for their wrinkle spatial distribution by Infinite Focus Microscopy (IFM) in an Alicona equipment (Bruker, Leicestershire, UK) that allowed us to evaluate the wavelength (λ) and the amplitude (A) of the wrinkles. In each surface, three images, 200 × 200 µm^2^, were acquired, and the roughness parameters were evaluated at two different locations. The nano-topography of the deposited PA thin films was studied by Atomic Force Microscopy (AFM)) in both PDMS and Si substrates. The characterization was performed in a diInnova equipment, from Veeco (Barcelona, Spain), in tapping mode. Si tips (Bruker, Leicestershire, UK) with a resonance frequency (f_0_) of 150 kHz and a spring constant (k) of 10N m^−1^ were used. The images were treated in Gwyddion^®^ software (version 2.0). For each surface, three different areas were scanned in order to ensure that the observed nano-topography was representative of the surface. The morphology of both surface and cross-sections of the thin films deposited on PDMS and Si were observed by Scanning Electron Microscopy (SEM), using a FEI Quanta 400FEG ESEM/EDAX Genesis X4M equipment (FEI, Hillsboro, Oregon, EUA).

### 2.3. Structural Characterization

X-Ray Diffraction (XRD)) was used to study the structural order of the deposited thin films. An X’Pert micro-diffractometer, from Panalytical (Kassel, Germany), with a Copper anti-cathode (λ_Kα_ = 0.15418 nm), was used. The diffractograms were collected using an accelerating voltage of 45 kV, 40 mA current, a scan from 10 to 60° in 2*θ*, with a step of 0.03° and a time per step of 1 s.

### 2.4. Chemical Analysis 

The X-ray Photoelectron Spectroscopy (XPS) study was performed in a Kratos Axis Ultra HSA equipment (Kratos Analytical, Manchester, UK, using an X-ray Al Kα monochromatic beam (λ = 0.83401 nm) with a tension of 15 kV. The analysis was performed with the beam perpendicular to the surface, and the data analyzed using CasaXPS^®^ software (version 2.3.20, Casa Software, Lda).

### 2.5. Wettability and Surface Charge

The evaluation of the surface wettability was made using the static contact angle measurements, using water and formamide. A drop of 10 µL of liquid was placed on each surface, and the contact angle measured by a DataPhysics QCA-20 equipment (DataPhysicsFilderstadt, Germany) at 25 °C. On each surface, a minimum of seven measurements were made.

The surface charge was evaluated through the zeta potential values. These were determined in SurPASS equipment, from Anton Paar GmbH (Graz, Austria). KCl 1mM, pH 7.4, which was used as the electrolyte, and the samples placed in the clamping cell system. For each surface, a minimum of 8 measurements were made.

### 2.6. Mechanical Properties 

The mechanical properties of PDMS and N1/PDMS were assessed in an Autograph AGS-1kNX model from Shimadzu (Kyoto, Japan), and the TRAPEZIUM X program (Shimadzu, Kyoto, Japan) was used for the treatment of the results. All tests were made in triplicate at room temperature, 25 °C, with a relative humidity of 30%. Samples with a length of 100 mm and 30 mm width were used in the tensile tests, and the load applied at a loading speed of 5 mm.min^−1^ until the rupture of the material.

Due to the viscoelastic nature of the polymeric materials, the loading and unloading curves do not coincide and, instead, a hysteresis loop is formed, which is a measure of the energy lost through heat transfer mechanisms during the deformation. In order to evaluate the influence of the deposited N1 coating on the properties of PDMS, cyclic tests were conducted by subjecting the samples to 10 cycles. In each one, the load was applied until it reached 20% of sample strain, with a loading speed of 7.5 mm.min^−1^. A preload of 2 N was applied to each specimen to ensure that all were strained at the same level at the beginning of the test.

### 2.7. Microbial in Vitro Tests 

In this study, four bacterial strains were used: two Gram-negative (Gram-) *Escherichia coli* (*E. coli*), *Pseudomonas aeruginosa* (*P. aeruginosa*), and two Gram-positive (Gram+) *Staphylococcus aureus* (*S. aureus*) and *Bacillus subtilis* (*B. subtilis*), all from the University of Coimbra Bacteria Culture Collection (Coimbra, Portugal). Before the tests, PDMS and Si substrates, unmodified and coated with PA thin films, were placed in the airflow chamber in a multiwall with 2 mL of ethanol solution (70% (v/v)) for 30 min, for sterilization. Luria–Bertani (LB) exponential phase cultures of the bacterial strains were diluted with sterile LB growth medium to an optical density (OD600nm) of 0.1.

Sterile LB growth medium was prepared with agar and the solution placed in Petri dishes. After this, 1 mL of bacterial suspension (0.1 OD600) was placed over the solid medium and spread with a glass spreader. The sterile modified and unmodified substrates were placed over the inoculated solid medium, with the surface of interest facing down. All the inoculated Petri dishes were incubated at 30 °C for 65 h. The tests were repeated twice in duplicate.

All the tested surfaces were chemically fixed and dehydrated, as previously described [24]. The surfaces were briefly washed three times with deionized water and were placed in multiwell plates. An amount of 3 mL of a glutaraldehyde solution (2.5%, v/v) was placed in each well for 10 min. After thorough washing with deionized water, the surfaces were subject to dehydration by placing in each well ethanol:water solution for 10 min, with increasing concentration of the alcoholic phase: 25%, 50%, 75%, and 100%. After drying, the surfaces were coated with 10 nm of sputtered gold to allow their observation in SEM.

## 3. Results and Discussion

### 3.1. Preliminary Study

**Figure 1:**
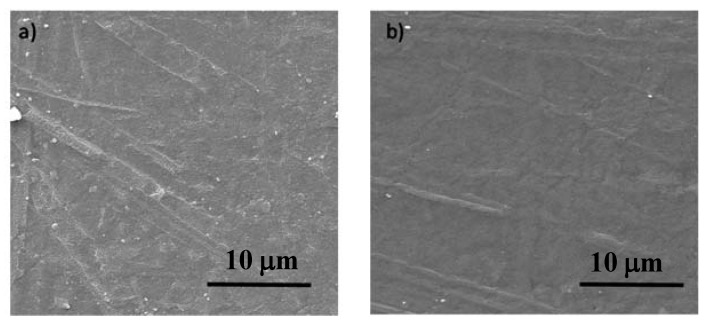
Scanning Electron Microscopy (SEM) micrographs of the PDMS surface: (**a**) pristine material; (**b**) after plasma cleaning with 250 W.

The first characterization was made by SEM in order to understand whether the wrinkle formation was due to the cleaning or the deposition process. The SEM micrographs of Figure 1 indicate that after the cleaning process, no wrinkles were visible on the PDMS surfaces.

This result is in contradiction with previous work by other authors [23] that reported that wrinkle formation is due to the removal of carbon from PDMS during the cleaning process, thus creating silica like surface, which, due to the development of compressive stress, leads to the wrinkle formation. The reported work claimed that this process is independent of the coating deposition step. However, in the present work, SEM characterization shows that, under the used experimental conditions, the wrinkle formation is not related to this mechanism as no wrinkles were observed after the cleaning step.

**Figure 2:**
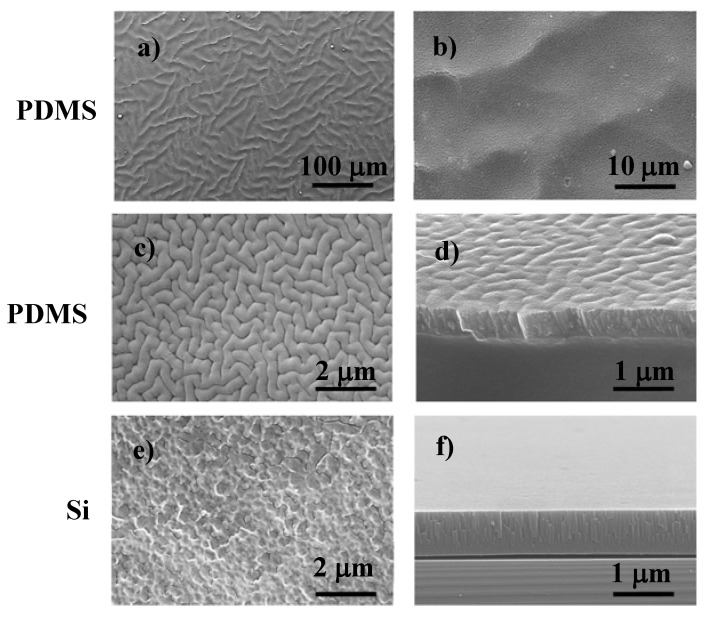
SEM micrographs of poly(amide) (PA) thin film deposited on PDMS: (**a**) N3, (**b)** N2, (**c**) N1 surface, and (**d**) N1 cross-section morphology. The same thin film (N1) deposited onto Si: (**e**) surface and (**f**) cross-section morphology.

After the deposition of the PA thin film deposited onto PDMS and Si substrates, in the same batch, different surface topographies were obtained as observed by SEM (Figure 2). The difference in the thickness of the coatings depending on the substrate material was expected, especially when considering that PDMS is electrically insolating while Si is a semiconductor material [25]. 

Moreover, it should be highlighted that, in thin films, intrinsic stress is more pronounced closer to the substrate, due to lattice mismatch [26]. This fact is evident in the cross-section micrograph of the PA thin film on Si (Figure 2f), where the tension applied during the fracture of the substrate/coating system induced the release of the interface tensions leading to the detachment of the coating. On the PDMS coated substrate, no lack of adhesion was observed due to the chemical compatibility between the polymeric material of the substrate and the polymeric material of the thin film. Therefore, the adhesion forces are higher than the cohesive ones in opposition to what occurs in the PA thin films deposited onto Si.

**Figure 3:**
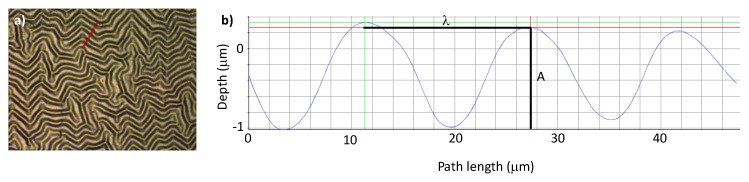
Infinite Focus Microscopy (IFM) of N1 thin film deposited onto PDMS: (**a**) optical image; (**b**) 2D profile over 50 µm (red line in a)) enabling wrinkle wavelength (λ) and amplitude (A) determination. (In b) red line correspond to the measured length, green lines were used to evaluate amplitude, and blue line is the 2D profile of the surface).

In order to characterize the roughness parameters at the micro-scale, the thin films were analyzed by IFM (Figure 3). This characterization enabled determining the surface roughness parameters as well as the wrinkle wavelength (λ) and amplitude (A) (Table 1).

From the results in Table 1, it is possible to observe that there is a relation between wavelength and amplitude of the wrinkles and the power used during the cleaning process, which can be justified by the increase of the substrate temperature as a consequence of its bombardment by a higher number of particles, with more energy, as the applied power increases, thus inducing the additional expansion of the polymeric substrate [25]. After the deposition of the film, the cooling stage induced the formation of wrinkles with a longer wavelength and higher amplitude in the substrates that reached higher temperatures. 

Accordingly, the average surface roughness (Sa), and the average surface quadratic roughness (Sq) increases with higher spatial dimensions of the wrinkles. The dimensionless parameter skewness measures the symmetry of the profile, in relation to the median plane, thus comparing the distribution and height of the peaks with the depth of the valleys. A symmetric profile has a skewness value of zero. Kurtosis is another dimensionless parameter and evaluates the “smoothness” of the surface. A value of three defines the limit, and the surface is considered flattened (<3) or sharped (>3), respectively, having a platikurtic or leptokurtic distribution, respectively [27]. 

According to the surface roughness parameters, the N1 surface was selected to pursue the rest of the characterization and the in vitro tests. By selecting this surface (with the lowest λ and A values), we intended to hinder the adhesion of the prokaryotic cells. Therefore, the chemistry of the surface must be appealing in order for the adhesion step of the bacterial colonization to occur and be irreversible, therefore evaluating the real influence of the topography. A negative value of the skewness parameter indicates that, although with a very symmetric distribution, the valleys present some predominance over the peaks.

### 3.2. Surface Characterization

**Figure 4:**
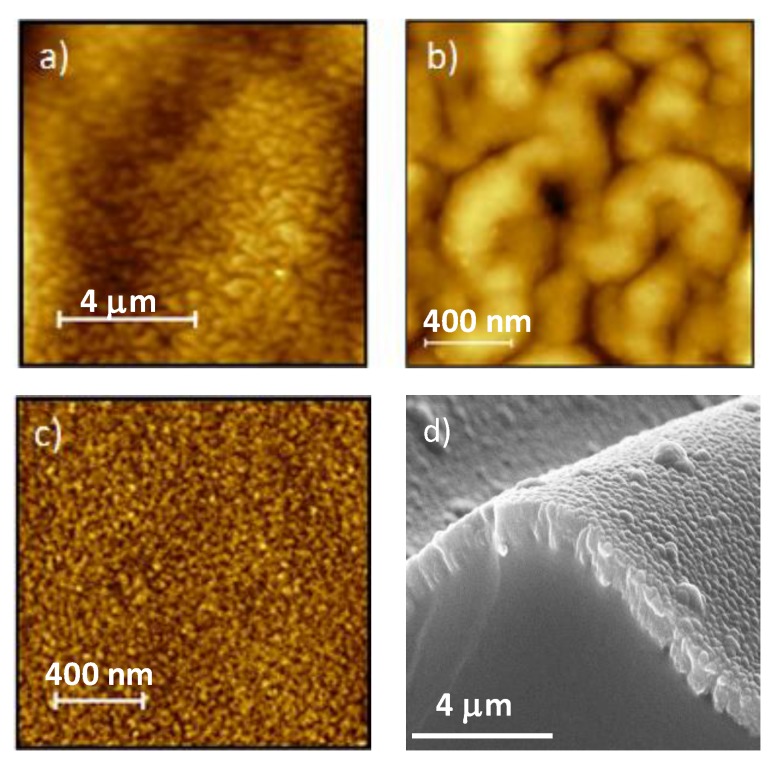
AFM topographic images of N1 surface deposited onto: (**a**), (**b**) PDMS, and (**c**) Si. (**d**) SEM micrograph of double scale wrinkles of N1.

A more detailed topographic characterization was made by AFM and observed by SEM (Figure 4) for the N1 surface deposited both onto PDMS and Si. It is visible the double scale roughness of the thin film deposited onto the PDMS substrate (Figure 4a,d). The roughness parameters were determined, for both substrates, in the same 2 × 2 µm^2^ area (Figure 4b,c) and the values presented in Table 2.

The structural characterization of both pristine targets and N1 thin film was made through XRD (diffractograms not shown). The target material presented a diffractogram typical of a semi-crystalline polymer, and the diffraction peaks were identified as polyamide according to reference pattern nº 043-1661 from the International Center for Diffraction Data (ICDD). As expected, the structural order of the bulk target is lost during the sputtering of the polymer, regardless of the substrate used, a known fact for supper-deposited polymeric materials [28], due to chemical rearrangement of the ejected molecular species. When these arrived at the substrate, their diffusion and ordered structural arrangement is hindered by the size of the aggregates, and amorphous coatings are produced. 

**Figure 5:**
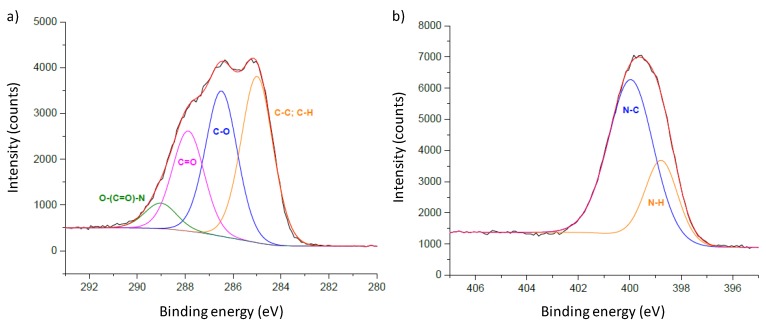
The X-ray Photoelectron Spectroscopy (XPS) Carbon (**a**) and Nitrogen (**b**) high-resolution spectra of N1 thin film deposited onto Si.

The chemical composition of the deposited polymer was evaluated by XPS (Figure 5). The chemical elements detected where carbon (C), oxygen (O), and nitrogen (N), as expected, with average atomic percentages of 57%, 11%, and 31%, respectively. The deconvolution of the high-resolution spectra of C and N (Figure 5) indicates the presence of the expected chemical bonds, with particular emphasis on the amide group (R–(CO)–N), characteristic of polyamide. 

We considered that the interface between abiotic material and prokaryotic cells, surface wettability, and charge also plays an essential role in the performance of the developed material. For the N1 PA thin film, the static contact angles with water of the films deposited onto Si and PDMS and the zeta potential values are displayed in Table 3.

The values of the contact angle are different for the film deposited onto different substrates. This result was predictable, since roughness is one of the parameters that has a significant influence on the wettability. However, it was not expected that the value of the coating onto PDMS was higher than the one determined when the substrate is Si. In fact, according to the Wenzel equation [29], if a hydrophilic surface is roughened, it becomes more hydrophilic. Therefore, the contact angle of water on the N1/PDMS surface should be lower than 59° and not around 100°. This apparent contradiction is related to the capability of a liquid to flow through a rough solid [30]. 

**Figure 6:**
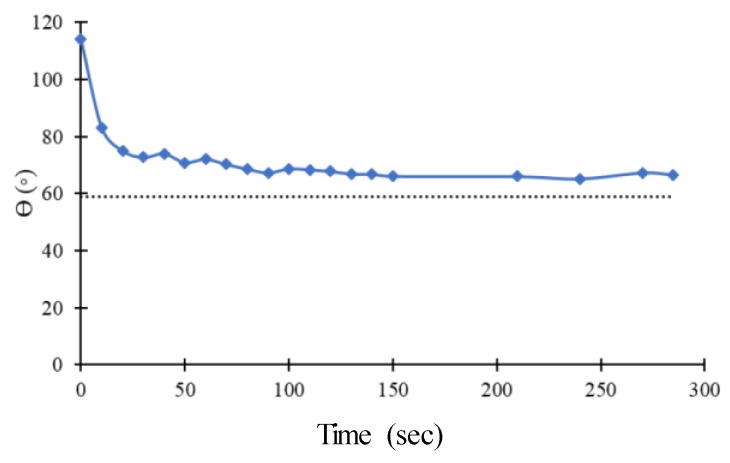
Variation, with time, of the contact angle of a drop of water onto the surface of double scale wrinkled N1 coated PDMS. The dotted line indicates the average value of the contact angle of the same coating onto Si substrate.

The measurement of the contact angle with time (Figure 6) shows that, after allowing the liquid through its chemical interaction with the surface to overcome the topography, the value is very similar to the contact angle obtained in the same thin film sputter-deposited onto Si. The same result was also observed by other authors that, instead of measuring the contact angle over time, applied strain in order to promote drop movement in the sliding mode [31].

The surface energy (γ_s_) was calculated according to the Young–Dupre equation as described previously [28] and presented as the sum of its polar (γ_s_^p^) and dispersive (γ_s_^d^) components (Table 3). The determination of the surface energy of the modified surfaces (Table 3), in particular by the polar component, γ_s_^p^, shows that it actively contributes to the wettability of the surfaces and is directly related to the presence of hydrophilic functional chemical groups on the surface as observed in XPS analysis. In addition, a surface with a surface energy of 50 mJ.m^−2^ is considered mildly reactive [32], which is in accordance with the reactivity of the chemical functional groups presented in the outermost surface, as determined by XPS.

The zeta potential of the surfaces is negative (Table 3), as was expected for surfaces that present a high content of –C–O groups such as carboxylic and carbonyl groups [28]. Only the presence of amine groups could decrease the negative value of the surface charge, but the nitrogen present in the sample is in an amide group and, therefore, it does not balance the negative charge of the surface [28]. The determined value is out of the range were surfaces are considered unstable, +30 to −30 mV. Therefore, consistent behavior is expected from these surfaces when exposed to high ionic strength environments such as the one in the culture media and, therefore, the interaction of the prokaryotic cells must be conditioned by the topographic cues.

The mechanical properties determined by tensile testing, Young’s modulus (for 1% strain, E_1_), the toughness (U_t_) and the rupture stress (σ_r_), and strain (ε_r_) at rupture, are all summarized in Table 4. 

Although the thickness of the thin film is minimal when compared to the substrate, the mechanical properties of the modified PDMS are different from the original PDMS. These results are not surprising, as PA coating has different mechanical properties than PDMS, and is the core of the wrinkle formation mechanism, as presented previously. Therefore, the presence of the coating confers more stiffness to the PDMS due to the presence of unsaturated bonds and cross-linked chains that are formed during the sputtering process of polymers [24].

**Figure 7:**
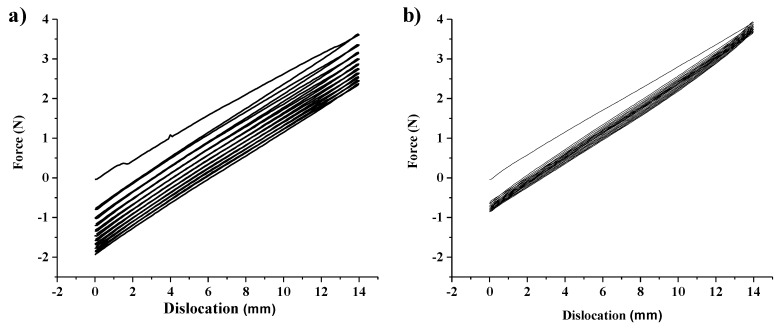
Loading-unloading curves obtained from the cyclic loading tests for: (**a**) PDMS; (**b**) N1 coated PDMS.

The area enclosed by the curves obtained during the cycling loading tests (Figure 7), the hysteresis loop area, represents the energy absorbed in each cycle. For a viscoelastic material, some of the strain energy is stored as potential energy, and some dissipates as heat. Once the applied load is removed, the potential energy stored is available for the body to recover some of the deformations. 

However, there is not enough energy to return to its original configuration [33]. The value of each hysteresis energy is a convenient measure of the accumulated fatigue damage [34]. The energy values, for the first and tenth cycles of each sample, are summarized in Table 4, where it is possible to confirm the decrease of their value with the number of cycles. Besides the fact that the first cycle corresponds to higher energy in the uncoated PDMS, it also clearly seems that the N1 coated sample has very low energy in each of the subsequent cycles, while PDMS shows a progressive decrease but always has higher values than the ones registered for the modified elastomer [35]. These results show that the presence of the PA thin film induces a more stable mechanical performance of the coating/substrate than the original PDMS [36]. 

After these characterizations, it was demonstrated that the significant difference between the polymeric surfaces deposited onto N1-PDMS and N1-Si is their topography. Moreover, it is topography that is likely to occur on medical devices and other related equipment that exists in hospital and related facilities, unlike the holes, pillars, and other engineered surfaces that are often reported in literature for the study of the topographic effect of surfaces on bacterial adhesion.

### 3.3. Microbial in Vitro Tests

**Figure 8:**
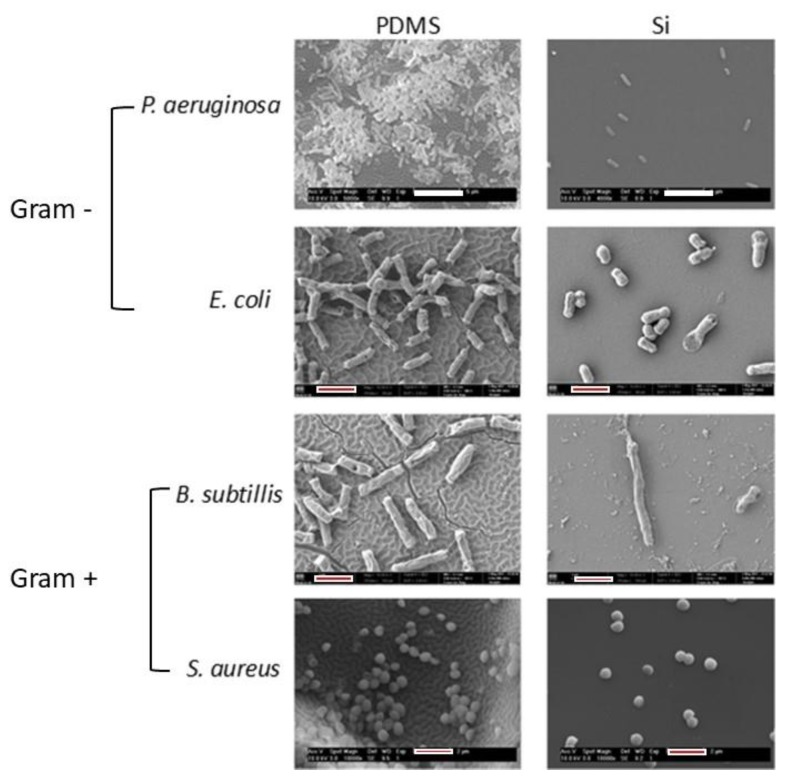
SEM micrographs of the N1 thin film deposited onto PDMS and Si after the growth-halo inhibition test (white bars = 5 µm; red & white bars = 2 µm).

The in vitro tests were performed with Gram- strains *E. coli* and *P. aeruginosa* and Gram+ bacterial strains from the species *B. subtilis* and *S. aureus*. The inhibition growth on solid media tests did not result in any inhibitory halo formation. The chemistry of the deposited surfaces presents chemical groups, which do not induce any oxidative stress to the bacteria [37] or eukaryotic cells [38] and, therefore, no inhibition halo was expected to occur [39,40]. Moreover, by choosing a test where the surfaces are facedown when in contact with the bacteria ensures that the preference to valleys or peaks is not due to gravity-driven cell settlement. This fact allows us to discuss the effect of topography independent of surface chemistry. The observation of bacterial surface colonization of the samples, after incubation, was performed by SEM (Figure 8).

In order to integrate and discuss the results, it should be noticed that besides being Gram+ and Gram-, the studied bacteria present other relevant differences. All the bacterial strains are rod except for the spherical *S. aureus*. Moreover, all the rod-shaped bacteria are flagellated, while the spherical one is not. Within the flagellated species, some differences can be summarized, namely: *B. subtillis* are eximious users of the flagella for locomotion when compared with the other two species, and from these two *P. aeruginosa* has a higher number of flagella than *E. coli*. All these differences influence the results as it must be considered not only the non-active result, which is due to the properties/characteristics of the abiotic surface, but especially the active metabolic response that is due only to the cell’s response to the abiotic surface. The interaction of the microorganism with the abiotic surface induces changes in the expression of genes that, consequently, influences cell morphology, mobility, and surface attachment [41]. 

The first observation shows that every bacterial strain prefers the hierarchical wrinkled surface over the nano-scale roughness. The results indicate that the chemical composition of the bacterial wall, the shape of the microorganism, or the presence of flagella cannot be considered differentiating features. Overall, all the bacteria cover a higher fraction area in the double-scale wrinkled surface than in the one that presents only nano-topographic features. This result is very interesting, considering that the coating over the Si substrate has a higher surface contact area with the solid culture medium inoculated with the microorganisms than the same thin film over PDMS.

Furthermore, considering that in the surfaces with hierarchical topography the valleys of the surface do not directly contact the solid medium, the results from *P. aeruginosa* and *S. aureus* indicate that besides adhesion, proliferation occurred and the prokaryotic cells “migrated” to the deeps presented in the surface.

The obtained results with prokaryotic cells are in agreement with other works reported in literature concerning eukaryotic cells. In recent years, the influence of topographical clues on decisions of the behavioral aspects of the cell has been gaining relevance and, currently, are considered as one of the main factors that influence cellular behavior. Cells can adapt their shape and orientation depending on the topography of the surface where they are placed. This phenomenon was studied initially by Weiss, who designed it as contact guidance [42,43,44]. This cellular orientation is explained through mechanodetection cycles, mechanotransduction, and mechanoresponse that occur in the cell and allows them to evaluate the surface topography and define a response according to its interpretation [45].

Topography at the nanometric scale influences the cell behavior in a more fundamental way, namely in the organization of the receptors that promote the adhesion of the cells, the integrins. The micrometric scale has more influence on cellular and supracellular characteristics, such as cell morphology and migration processes [43,44,46]. Studies showed that the adhesion process is strictly dependent on both the nanometric and micrometric scales. In other words, nano-topography influences agglomeration of integrins; however, the arrangement and maturation of focal adhesion (junctions that allow cell/substrate bonding) are influenced by micro-tography [35]. Therefore, a topography with a combination of the two scales is more favorable than the use of each one separately.

## 4. Conclusions

In this work, the real effect of the surface topography in the adhesion of the different bacterial strains Gram+ and Gram-, with rod and spherical morphology, with and without flagella, was evaluated. An attractive surface chemistry was chosen by depositing polyamide thin films onto two different substrates, PDMS and Si, inducing hierarchical topography and nano-scale features, respectively. The power density used for the plasma cleaning of PDMS, which was previously used for the thin film deposition, induced differences in the Sa, λ, and A surface parameters of the produced hierarchical wrinkles. 

The comparison of the adhesion and proliferation of the prokaryotic cells was made between the surface with nanotopographic features (Sa ≈ 1 nm) and the hierarchical wrinkles of the N1 surface (Sa = 750 nm, λ = 16 µm, and A = 1 µm). The results indicate that when bacteria interact with an abiotic surface with amicable surface chemistry, a double-scale topography that mimics the extracellular hierarchical matrix is preferred. 

## Figures and Tables

**Table 1:** Wavelength, amplitude, and micrometer roughness parameters, determined by IFM, of the PA thin films deposited onto PDMS substrates.

Thin Film	λ (µm)	A (µm)	Roughness Parameters			
			Sa (nm)	Sq (nm)	*Skewness*	*Kurtosis*
N1	15.9 ± 0.3	1.2 ± 0.2	754 ± 30	963 ± 5	−0.25	3.10
N2	30.0 ± 10	2.7 ± 0.8	353 ± 50	440 ± 10	0.30	2.92
N3	28.6 ± 1.6	3.1 ± 0.5	1006 ± 45	1201 ± 20	--	--

**Table 2:** Roughness parameters, determined by AFM, of N1 thin film deposited onto PDMS and Si substrates.

Substrate	Roughness Parameters				
	Sa (nm)	Sq (nm)	Sz (nm)	*Skewness*	*Kurtosis*
Si	0.91	1.7	6.40	0.09	−0.11
PDMS	24.5	31.1	226.2	−0.44	0.52

**Table 3:** Static contact angles, surface energy, and zeta potential of N1 deposited thin film.

	Contact Angle(°)		Surface Energy (mJ.m^−2^)		
Substrate	Water	Formamide	γ_S_^d^	γ_S_^p^	Zeta Potential (mV)
Si	59 ± 3	32 ± 2	37.1	12.9	−52.8 ± 15.4
PDMS	100 ± 3	---	---	---	

**Table 4:** Mechanical properties of PDMS and N1 coated PDMS.

				Energy Loss (J.m^−3^)	
	E_1_ (MPa)	σ_r_ (MPa)	ε_r_ (%)	1^st^ cycle	10^th^ cycle
N1	1.24 ± 0.05	1.56 ± 0.3	115 ± 8	4320	325
PDMS	0.55 ± 0.17	1.77 ± 0.09	189 ± 14	3730	210

## References

[1] Christo S.N., Bachhuka A., Diener K.R., Mierczinska A., Hayball J.D., Vasilev K. (2016). The role of surface nanotopography and chemistry on primary neutrophil and macrophage cellular responses. Adv. Healthc. Mater..

[2] Li M., Joung D., Hughes B., Waldman S.D., Kozinski J.A., Hwang D.K. (2016). Wrinkling non-spherical particles and its application in cell attachment promotion. Sci. Rep..

[3] Wang Y.L., Deng J.J., Fan R.R., Tong A.P., Zhang X.N., Zhou L.X., Zheng Y., Xua J.G., Guo G. (2015). Novel nanoscale topography on poly(propylene carbonate)/poly(ε-caprolactone) electrospun nanofibers modifies osteogenic capacity of ADCs. RSC Adv..

[4] Zou F., Zhou H., Jeong D.Y., Kwon J., Eom S.U., Park T.J., Hong S.W., Lee J. (2017). Wrinkled surface-mediated antibacterial activity of graphene oxide nanosheets. ACS Appl. Mater. Interfaces.

[5] Alves N.M., Pashkuleva I., Reis R.L., Mano J.F. (2010). Controlling cell behavior through the design of polymer surfaces. Small.

[6] Piedade A.P., Gil M.H., Cavaco M.C., Andrade M.E. (1995). Behaviour of catalase immobilised on poly(acrylonitrile)-g.co-hydroxyethyl methacrylate when used in a continuous system. Polym. Int..

[7] DaSilva M.A., Gil M.H., Piedade A.P., Redinha J.S., Brett A.M.O., Costa J.M.C. (1991). Immobilization of catalase on membranes of poly(ethylene)-g.co-acrylic acid and poly(tetrafluoroethylene)-g.co-acrylic acid and their application in hydrogen peroxidase electrochemical sensors. J. Polym. Sci. A.

[8] Brett A.M.C.F.O., Gil M.H., Piedade A.P. (1992). An electrochemical bienzyme membrane sensor for free cholesterol. Bioelectrochem. Bioenerg..

[9] Rodríguez-Hernández J. (2015). Wrinkled interfaces: Taking advantage of surface instabilities to pattern polymer surfaces. Prog. Polym. Sci..

[10] Zhou Q., Kühn P.T., Huisman T., Nieboer E., van Zwol C., Kooten T.G., van Rijn P. (2015). Directional nanotopographic gradients: A high-throughput screening platform for cell contact guidance. Sci. Rep..

[11] Cheng Y., Feng G., Moraru C.I. (2019). Micro- and nanotopography sensitive bacterial attachment mechanisms: A review. Front. Microbiol..

[12] Erramilli S., Genzer J. (2019). Influence of surface topography attributes on settlement and adhesion of natural and synthetic species. Soft Matter.

[13] Rigo S., Cai C., Gunkel-Grabole G., Maurizi L., Zhang X., Xu J., Palivan C.G. (2018). Nanoscience-based strategies to engineer antimicrobial surfaces. Adv. Sci..

[14] Li B., Cao Y.P., Feng X.Q., Gao H. (2012). Mechanics of morphological instabilities and surface wrinkling in soft materials: A review. Soft Matter.

[15] Adam A., Paulkowski D., Mayer B. (2019). Friction and deformation behavior of elastomers. Mater. Sci. Appl..

[16] Piedade A.P., Nunes J., Vieira M.T. (2008). Thin films with chemically graded functionality based on fluorine polymers and stainless steel. Acta Biomater..

[17] Piedade A.P., Pinho A.C., Branco R., Morais P.V. (2020). Evaluation of antimicrobial activity of ZnO based nanocomposites for the coating of non-critical equipment in medical-care facilities. Appl. Surf. Sci..

[18] Piedade A.P., Vieira M.T., Martins A., Silva F. (2007). In vitro behaviour of nanocrystalline silver-sputtered thin films. Nanotechnology.

[19] Piedade A.P., Dias D., Branco R., Morais P.V. (2020). Unsaturated carbon linear chains created during bacteria incubation with amorphous carbon thin films produced by a clean technology. J. Clean. Prod..

[20] Li S.J., Wu K., Yuan H.Z., Zhang J.Y., Liu G., Sun J. (2019). Formation of wrinkled patterns in metal films deposited on elastic substrates: Tunability and wettability. Surf. Coat. Technol..

[21] Yu S., Ma L., Sun Y., Lu C., Zhou H., Ni Y. (2019). Controlled wrinkling patterns in periodic thickness-gradient films on polydimethylsiloxane substrates. Langmuir.

[22] Serrano J.R., Xu Q., Cahill D.G. (2006). Stress-induced wrinkling of sputtered SiO_2_ films on polymethylmethacrylate. J. Vac. Sci. Technol. A.

[23] Casper M.D., Gözen A.Ö., Dickey M.D., Genzer J., Maria J.P. (2013). Surface wrinkling by chemical modification of poly(dimethylsiloxane)-based networks during sputtering. Soft Matter.

[24] Carvalho D., Sousa T., Morais P.V., Piedade A.P. (2016). Polymer/metal nanocomposite coating with antimicrobial activity against hospital isolated pathogen. Appl. Surf. Sci..

[25] Greene J.E. (2017). Tracing the recorded history of thin-film sputter deposition: From the 1800s to 2017. J. Vac. Sci. Technol. A.

[26] Furlan A., Grochla D., D’Acremont Q., Pernot G., Dilhaire S., Ludw A. (2017). Influence of substrate temperature and film thickness on thermal, electrical, and structural properties of HPPMS and DC magnetron sputtered Ge thin films. Adv. Eng. Mater..

[27] Gadelmawla E.S., Koura M.M., Maksoud T.M.A., Elewa I.M., Soliman H.H. (2002). Roughness parameters. J. Mater. Process. Technol..

[28] Pinho A.C., Piedade A.P. (2013). Zeta potential, contact angles, and AFM imaging of protein conformation adsorbed on hybrid nanocomposite surfaces. ACS Appl. Mater. Interfaces.

[29] Wenzel R.N. (1936). Resistance of solid surfaces to wetting by water. Ind. Eng. Chem..

[30] Quéré D. (2002). Rough ideas on wetting. Phys. A.

[31] Lin G., Chandrasekaran P., Lv C., Zhang Q., Tang Y., Han L., Yin J. (2017). Self-similar hierarchical wrinkles as a potential multifunctional smart window with simultaneously tunable transparency, structural color, and droplet transport. ACS Appl. Mater. Interfaces.

[32] Vogler E.A. (1998). Structure and reactivity of water at biomaterial surfaces. Adv. Colloid Interface Sci..

[33] Ozkaya N., Leger D., Goldsheyder D., Nordin M. (2016). Fundamentals of Biomechanics: Equilibrium, Motion, and Deformation.

[34] Kliman V., Bily M. (1984). Hysteresis energy of cyclic loading. Mater. Sci. Eng..

[35] Kim T.K., Kim J.K., Jeong O.C. (2011). Measurement of nonlinear mechanical properties of PDMS elastomer. Microelectron. Eng..

[36] Kim J.H., Hwang J.-Y., Hwang H.R., Kim H.S., Lee J.H., Seo J.-W., Shin U.S., Lee S.-H. (2018). Simple and cost-effective method of highly conductive and elastic carbon nanotube/polydimethylsiloxane composite for wearable electronics. Sci. Rep..

[37] Moshynets O., Bardeau J.-F., Tarasyuk O., Makhno S., Cherniavska T., Dzhuzha O., Potters G., Rogalsky S. (2019). Antibiofilm activity of polyamide 11 modified with thermally stable polymeric biocide polyhexamethylene guanidine 2-naphtalenesulfonate. Int. J. Mol. Sci..

[38] Piedade A.P., Veneza C., Duarte C.B. (2019). Polyamide 6.6 thin films with distinct ratios of the main chemical groups: Influence in the primary neuronal cell culture. Appl. Surf. Sci..

[39] Ferraris S., Perero S., Miola M., Vernè E., Rosiello A., Ferrazzo V., Valletta G., Sanchez J., Ohrlander M., Tjörnhammar S. (2008). Chemical, mechanical and antibacterial properties of silver nanocluster/silica composite coated textiles for safety systems and aerospace applications. Appl. Surf. Sci..

[40] Muñoz-Bonilla A., Echeverria C., Sonseca A., Arrieta M.P., Fernández-García M. (2019). Bio-based polymers with antimicrobial properties towards sustainable development. Materials.

[41] Tuson H.H., Wiebel D.B. (2013). Bacteria-surface interactions. Soft Matter.

[42] Weiss P., Hiscoe H.B. (1948). Experiments on the mechanism of nerve growth. J. Exp. Zool..

[43] Nguyen A.T., Sathe S.R., Yim E.K.F. (2016). From nano to micro: Topographical scale and its impact on cell adhesion, morphology and contact guidance. J. Phys. Condens. Matter.

[44] Nikkhah M., Edalat F., Manoucheri S., Khademhosseini A. (2012). Engineering microscale topographies to control the cell—Substrate interface. Biomaterials.

[45] Vogel V., Sheetz M. (2006). Local force and geometry sensing regulate cell functions. Nat. Rev. Mol. Cell Biol..

[46] Anselme K., Ploux L., Ponche A. (2010). Cell/material interfaces: Influence of surface chemistry and surface topography on cell adhesion. J. Adhes. Sci. Technol..

